# Non-toxigenic *Clostridioides* (Formerly Clostridium) *difficile* for Prevention of *C. difficile* Infection: From Bench to Bedside Back to Bench and Back to Bedside

**DOI:** 10.3389/fmicb.2018.01700

**Published:** 2018-07-26

**Authors:** Dale N. Gerding, Susan P. Sambol, Stuart Johnson

**Affiliations:** ^1^Research Service, Edward Hines Jr. VA Hospital, Hines, IL, United States; ^2^Department of Medicine, Loyola University Chicago Medical Center, Maywood, IL, United States

**Keywords:** non-toxigenic, *Clostridium difficile*, NTCD-M3, prevention, NTCD, colonization, spores, *Clostridioides difficile*

## Abstract

The beneficial effect of colonization of the gastrointestinal tract by non-toxigenic *Clostridioides difficile* (NTCD) strains as a preventive of toxigenic *C. difficile* infection (CDI) has been known since the early 1980s. Investigators in both the USA and United Kingdom demonstrated that prior colonization by randomly selected NTCD strains provided prevention against infection by toxigenic *C. difficile* in hamsters, albeit with limited durability. In the 1980s two patients with multiply recurrent CDI in the UK were treated with vancomycin followed by NTCD to prevent further recurrences, with one success and one failure. Epidemiologic studies of hospitalized patients using weekly rectal swab cultures demonstrated that asymptomatic colonization of patients by toxigenic *C. difficile* was much more common than CDI, but also that the rate of asymptomatic NTCD colonization of patients was unexpectedly high. Development of molecular strain typing of *C. difficile* was instrumental in characterizing different strains of both toxigenic *C. difficile* and NTCD leading to identification of NTCD strains that were effective human colonizers. These strains were reintroduced in hamsters in the 1990s and shown to prevent CDI efficiently and durably when challenged with epidemic toxigenic *C. difficile* strains. One strain of NTCD, NTCD-M3, was manufactured under cGMP standards and was demonstrated to be safe in a phase 1 volunteer trial. NTCD-M3 was then tested in a phase 2 double-blind placebo controlled trial for the prevention of recurrent CDI in patients experiencing their first CDI episode or first CDI recurrence. NTCD-M3 was given at doses of 10^4^ or 10^7^ spores per day orally for 7 or 14 days following successful treatment of CDI with vancomycin and/or metronidazole. CDI recurred in 30% of placebo patients and 11% of all NTCD-M3 patients (*p* = 0.006); recurrence rate for the best dose, 10^7^ spores/d × 7 days, was 5% (*p* = 0.01 vs. placebo). Detection of colonization predicted prevention success; among the 86 patients who were colonized with NTCD-M3 the recurrence rate was 2% vs. 31% in patients who received NTCD-M3 but were not colonized (*p* < 0.001). Additional trials of NTCD-M3 for primary prevention of CDI and prevention of CDI recurrence seem warranted by these promising results.

## Introduction

The purpose of this review is to summarize the experimental, pre-clinical, and clinical data for the use of non-toxigenic *Clostridioides difficile* (NTCD) for prevention of *C. difficile* infection (CDI) caused by toxigenic strains of *C. difficile*. NTCD strains in which the pathogenicity locus (PaLoc) for the main *C. difficile* virulence factors, toxin A and B is replaced by a 115 bp sequence occur naturally. Multiple investigators have published convincing results of experiments over the last 35 years that demonstrate the efficacy of NTCD as a colonizing bacterial agent that prevents infection with toxigenic strains of *C. difficile* in animals and humans. Although a definitive mechanism for this protection remains to be determined, the effectiveness of this approach in multiple animal models and human clinical observations and trials suggests that it is a safe and effective biotherapeutic strategy that could be used for both primary prevention of CDI and prevention of CDI recurrence.

### Background bench discoveries in the 1980s

The concept of NTCD inhibiting or interfering with the infectivity of a toxigenic strain of *C. difficile* was first reported by Wilson and Sheagren (1983) in the hamster model in 1983. They demonstrated that following cefoxitin administration, colonization with NTCD prior to challenge with toxigenic *C. difficile* increased hamster survival from 21% to 93%, however, durability of prevention was not determined. Simultaneous administration of the NTCD strain and a toxigenic *C. difficile* strain did not increase survival. Borriello and Barclay (1985) extended these observations by colonization of hamsters with three different strains of NTCD, M-1, D-1 and S-1 administered at doses ranging from 3 × 10^6^ to 3 × 10^9^ organisms followed by challenge 5 days later with a toxigenic strain B-1 at a dose of 3 × 10^7^ organisms. Results of these experiments showed at 25 days post toxigenic challenge survival of 13 of 18 (72%) vs. 0/21 (0%) hamsters not given an NTCD strain. Additional experiments showed (1) that protection was dependent upon live NTCD organisms; heat-killed NTCD did not protect hamsters, (2) that protection was dependent upon NTCD colonization; decolonization with vancomycin resulted in no protection against toxigenic challenge, and (3) that protection was specific to *C. difficile*; attempts to colonize with *C. perfringens, C. bifermentans*, and *C. beijerincki*, failed, and *C. sporogenes* colonized but failed to protect against toxigenic *C. difficile* challenge.

Borriello and Barclay (1985) also attempted to identify the mechanism by which NTCD protected against toxigenic *C. difficile*. They showed that NTCD did not degrade or alter the potency of *C. difficile* toxin. They investigated the mucosal association of NTCD by harvesting cecum samples from three NTCD-colonized hamsters at day 51 of colonization, washing them eight times with brain heart infusion (BHI) broth and saline, then homogenizing the tissue and doing quantitative culture of the homogenate. They found association of NTCD with the cecal mucosa ranging from 6 × 10^3^ to 3 × 10^5^ cfu/g tissue, suggesting there may be association of NTCD with the cecal mucosa or alternatively, they suggested competition for essential nutrients. However, in the discussion they report that after day 25 death occurred in most animals and that toxigenic *C. difficile* and cytotoxin could be detected in the cecum of these animals. Sequential analysis of the fecal pellets in these animals detected a change over time from only NTCD recovery to recovery of toxigenic *C. difficile* or both. This was of concern and the authors speculated that the presence of toxin enabled toxigenic strains of *C. difficile* to have a colonization advantage over NTCD. In a subsequent publication, Borriello et al. (1988) showed that a virulent toxigenic strain B-1 of *C. difficile* was more adherent to mucosa of small and large bowel in the hamster than NTCD strain M-1. They also showed that injection of toxin into the cecum of hamsters enhanced colonization of M-1 in the colon, but not in the small bowel or cecum when compared to toxigenic strain B-1.

Similar observations of protection against toxigenic *C. difficile* by NTCD were shown in gnotobiotic mice by Corthier and Muller (1988). NTCD protected against toxigenic challenge from 18h to 10 days following NTCD in 100% of gnotobiotic mice. If there was no delay in administration of the toxigenic strain following NTCD, 60% of mice survived. Survival of mice beyond 10 days was not recorded.

These early bench and animal studies were not further pursued beyond the 1980s, most likely because the protective effect in hamsters did not appear to be durable. However, these observations provide a valuable early insight into the potential utility of NTCD as a preventive strategy for CDI.

### Bedside clinical observations

The first published report of the use of NTCD in patients was by Seal et al. (1987). Two patients with multiple recurrences of CDI were treated orally with a BHI culture of NTCD strain M-1 previously shown by Borriello and Barclay (1985) to prevent CDI in hamsters transiently. One ml of the 24h culture was diluted in 50 ml of milk (final concentration ~1 × 10^7^ cfu/ml) which was administered once daily for 3 days. **Patient A**, age 88 years, was treated for her first CDI episode with metronidazole and for episodes two and three with oral vancomycin. Although successfully treated with resolution of symptoms, she had persistent *C. difficile* in her stool for at least 3 days following the last vancomycin treatment but was negative on stool culture just prior to receiving NTCD. Following NTCD her stool cultures were positive for NTCD for 14 days with no toxigenic *C. difficile* detected, and she had no further CDI recurrences for at least 4 months. **Patient B**, age 76, was also treated for her first CDI episode with metronidazole and for episodes two and three with vancomycin. She was treated successfully for her diarrhea symptoms with vancomycin, but had high counts of toxigenic *C. difficile* in her stool for the first 3 days of NTCD administration (5.9–7.5 log_10_/gm stool) with no NTCD detected. NTCD did not appear in the stool until day 6 at which time there were ~10^7^ cfu/gm stool of both NTCD and toxigenic *C. difficile*, but on days 9 and 10 only toxigenic *C. difficile* was found at a concentration of ~10^5^ cfu/gm stool. Starting on day 17 she had 2 days of self-limited diarrhea with both toxigenic *C. difficile* and NTCD at ~10^3^ cfu/gm stool and stool toxin detected. On day 28 counts of both toxigenic *C. difficile* and NTCD had declined to ~10^2^ cfu/gm and on day 45 neither were detected in stool. The patient died 1 month later of an unrelated cause but had no further diarrhea. In view of the mild recurrence of diarrhea with stool toxin detected at day 17 it is likely that this patient failed preventive treatment with NTCD, whereas patient A was a success. No further patient treatments with NTCD were reported until well into the 21^st^ century, however, considerable clinical information on detection of NTCD in patients was obtained in the interim.

In the 1980s it became apparent that CDI was a healthcare associated infection (Gerding et al., 1986; McFarland et al., 1989), and typing systems for identification of specific *C. difficile* strains were developed (Kuijper et al., 1987; McFarland et al., 1989; Clabots et al., 1993) based not only on isolates from patients with CDI, but also from specimens collected from asymptomatic patients using rectal swabs (McFarland et al., 1989; Johnson et al., 1990; Clabots et al., 1992). McFarland et al. (1989) were the first to detect the presence of large numbers of patients in hospital that were asymptomatically colonized with *C. difficile*. They demonstrated that transmission of *C. difficile* was frequent in the hospital setting using immunoblot organism typing and space/time relations. They found that nearly 2/3 of patients that acquired *C. difficile* remained asymptomatic whereas just over 1/3 had symptomatic CDI (McFarland et al., 1989).

The use of restriction endonuclease analysis (REA) typing of *C. difficile* markedly enhanced the epidemiologic investigation of CDI in hospitals (Kuijper et al., 1987; Clabots et al., 1993). Early in the development of REA there were 75 distinct groups identified by letters of the alphabet, 43 cytotoxin positive groups, 28 NTCD groups, and 4 groups that included both cytotoxin positive and negative isolates. Isolates for typing were obtained from CDI patients, rectal swabs of asymptomatic patients and multiple environmental sites. In comparison with other typing systems then in use REA demonstrated greater sensitivity than immunoblot typing, bacteriophage-bacteriocin typing and ribotyping (Clabots et al., 1993). Remarkably, the most frequently isolated REA group was the NTCD group M which was found more commonly than any toxigenic groups and may be a reflection of the inclusion of large numbers of isolates collected from asymptomatic patients using rectal swabs.

Using a similar weekly rectal swab methodology used by McFarland et al. (1989) to detect *C. difficile* asymptomatic colonization coupled with REA typing of *C. difficile* (Kuijper et al., 1987; Clabots et al., 1993), Johnson et al. (1990) showed that 21% of patients hospitalized on three wards were culture-positive for *C. difficile*, the majority 51 of 60 (85%) were asymptomatic. All of the 9 cases of CDI were caused by two closely related toxigenic REA types, B and B2, and none occurred among the 51 asymptomatic colonized patients, 9 of whom were colonized with the B or B2 strain types but remained asymptomatic. Fifteen of the 17 patients with the B or B2 isolates were located on the same surgical ward suggesting transmission on that ward. Among the 18 different REA type *C. difficile* isolates found on the 3 study wards, 10 were toxigenic and 8 (44%) were non-toxigenic including 7 distinct REA groups of NTCD, groups A, C, M, P, S, T, and U. This was the first indication that NTCD strains were carried frequently by hospital patients. In addition, it was found that asymptomatic *C. difficile* colonization occurred in approximately the same frequency on all 3 hospital wards, ranging from 12.2 to 19.3% of patients, all of whom had relatively long hospital stays. All the patients with toxigenic *C. difficile* or NTCD in their stool remained asymptomatic.

A larger similar study using rectal swab cultures was conducted on a single surgical ward with a historically high rate of CDI by Clabots et al. (1992). The study was conducted over 9 months and enrolled 634 (94%) of 678 admissions to this ward, obtaining rectal swab cultures on admission and weekly thereafter. Of the 634 ward admissions, 65 (10.3%) were either asymptomatically culture-positive for *C. difficile* (61 patients) or had CDI (4 patients). Twelve additional asymptomatic admissions were positive for *C difficile* when first sampled, but were not sampled within 48 h of admission and were indeterminate as to whether they acquired *C. difficile* on the ward or were already colonized when admitted. The majority of admissions (355, 56%) were admitted from home and had not been hospitalized within the previous 30 days. Twenty-two (7%) of these home admissions were culture-positive for *C. difficile* indicating the high potential for introduction of *C. difficile* strains to the hospital from the community. Of the 557 admissions to the ward that were not culture-positive for *C. difficile*, 54 (9.7%) acquired *C difficile* while on the ward, but only 3 had CDI. REA typing identified 58 different distinct types during the study. Of the top seven most frequently isolated REA types, three were NTCD types and constituted 23/68 (34%) of these isolates. The most commonly isolated NTCD strain type was M3 which subsequently, together with types M23 and T7, was selected for laboratory trials in hamsters for prevention of CDI.

**Figure 1:**
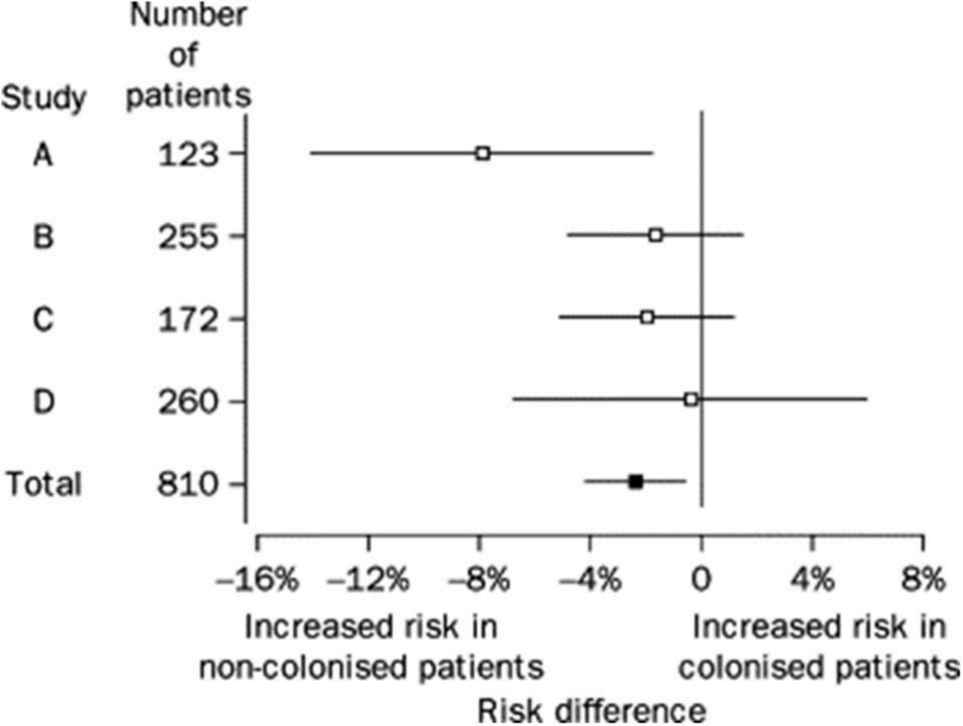
Risk difference (95% CI) for CDI among patients initially colonized and non-colonized with *C difficile* in four studies. Risk difference pooled across studies with a random effects model is shown in bottom row (Total). From Shim et al. (1998).

Low rates of CDI among patients previously asymptomatically colonized with *C. difficile* were observed in several trials utilizing weekly rectal swab cultures and suggested that colonization might be protective against CDI if symptoms did not develop relatively quickly following toxigenic *C. difficile* exposure, or if patients were colonized by NTCD. Data from four such prospective studies were combined in a random effects model by Shim et al. (1998). All four studies employed similar weekly rectal swab cultures for *C. difficile* in asymptomatic hospitalized patients and utilized stool cultures for *C. difficile* if CDI symptoms developed. Patients recovering from prior CDI were excluded as they have a high colonization rate with toxigenic *C. difficile* post treatment and are prone to recurrences of CDI. Asymptomatic patients were included if they had at least two consecutive weekly *C. difficile* rectal swab cultures and were classified as either colonized or not colonized. Colonized patients were further categorized as colonized with a toxigenic *C. difficile* strain or NTCD. All isolates underwent REA typing as well as toxin testing. Among the 618 non-colonized patients, 22 (3.6%) developed CDI whereas only 2 (1%) of 192 colonized patients developed CDI (pooled risk difference −2.3%, *p* = 0.021, Figure 1). When the analysis was confined only to patients who had received antibiotics within 14–21 days of the start of the sampling or during sampling, the pooled risk difference was −3.2%, *p* = 0.024. Greater exposure to antibiotics in non-colonized patients could explain the increased rate of CDI, but antibiotic exposure was lower (79%) in non-colonized patients compared to colonized patients (92%). The mechanism by which colonization prevents CDI is unknown, and toxigenic strains of *C. difficile* as typed by REA that caused CDI were also found to be carried asymptomatically suggesting that strain type did not explain prevention. In these 4 studies there was a very high proportion (44%) of patients that were colonized by NTCD. There were 15 different REA groups of NTCD found among the 76 NTCD isolates, but the majority of NTCD were in two REA groups, M (49%) and T (19%). No CDI occurred among NTCD colonized patients and may account at least partially for the reduced CDI rates in colonized patients.

Taken together these clinical observations documented the relatively high rate of colonization of hospitalized patients by non-toxigenic strains of *C. difficile* and documented a very low rate of CDI among all colonized patients. Furthermore, REA typing demonstrated the ability to group and type NTCD strains in a manner similar to toxigenic *C. difficile* and identified two dominant groups of NTCD, the M and T groups by REA typing.

### Back to bench investigation: pre-clinical NTCD development

Bedside clinical and epidemiologic observations coupled with the evolution of molecular typing of *C. difficile* prompted further laboratory testing of *C. difficile*, particularly in animal models. Of specific interest was whether strains of *C. difficile* responsible for hospital outbreaks were more virulent than other strains that were found to asymptomatically colonize patients more often than they caused CDI. Sambol et al. (2001) tested three toxigenic epidemic *C. difficile* strains, REA types B1, J9, and K14 in the hamster model and compared them to toxigenic REA type Y2 which was found to commonly colonize patients but caused little CDI. A fifth strain, toxin variant REA type CF2, which is toxin A-negative, toxin B-positive and associated with CDI outbreaks was also included. The hamster protocol was similar to that of Borriello and Barclay (Borriello and Barclay, 1985), but differed in that only spores of *C. difficile* were administered by gavage 5 days following a single oral clindamycin dose of 30 mg/kg. A minimum infective dose for each *C. difficile* strain was predetermined to be 100 spores to mimic a postulated low inoculum exposure. The epidemic strains B1, J9, and K14 infected hamsters within 24–48 h of administration and caused 100% mortality by 48 h. REA type Y2 infected 9 of 10 hamsters over a range of 1–4 days, somewhat more slowly than the epidemic strains, but eventually all 9 infected hamsters died. Toxin variant strain CF2 infected only 6 of 10 hamsters over a range of 1 to 10 days and only 3 of the 6 died; the remaining 3 hamsters were colonized through study termination at day 79 and appeared to have no symptoms. The study documented differences in virulence of *C. difficile* strains in the hamster model and served as a basis on which to test preventive strategies with NTCD in the hamster model.

#### NTCD prevention

**Figure 2:**
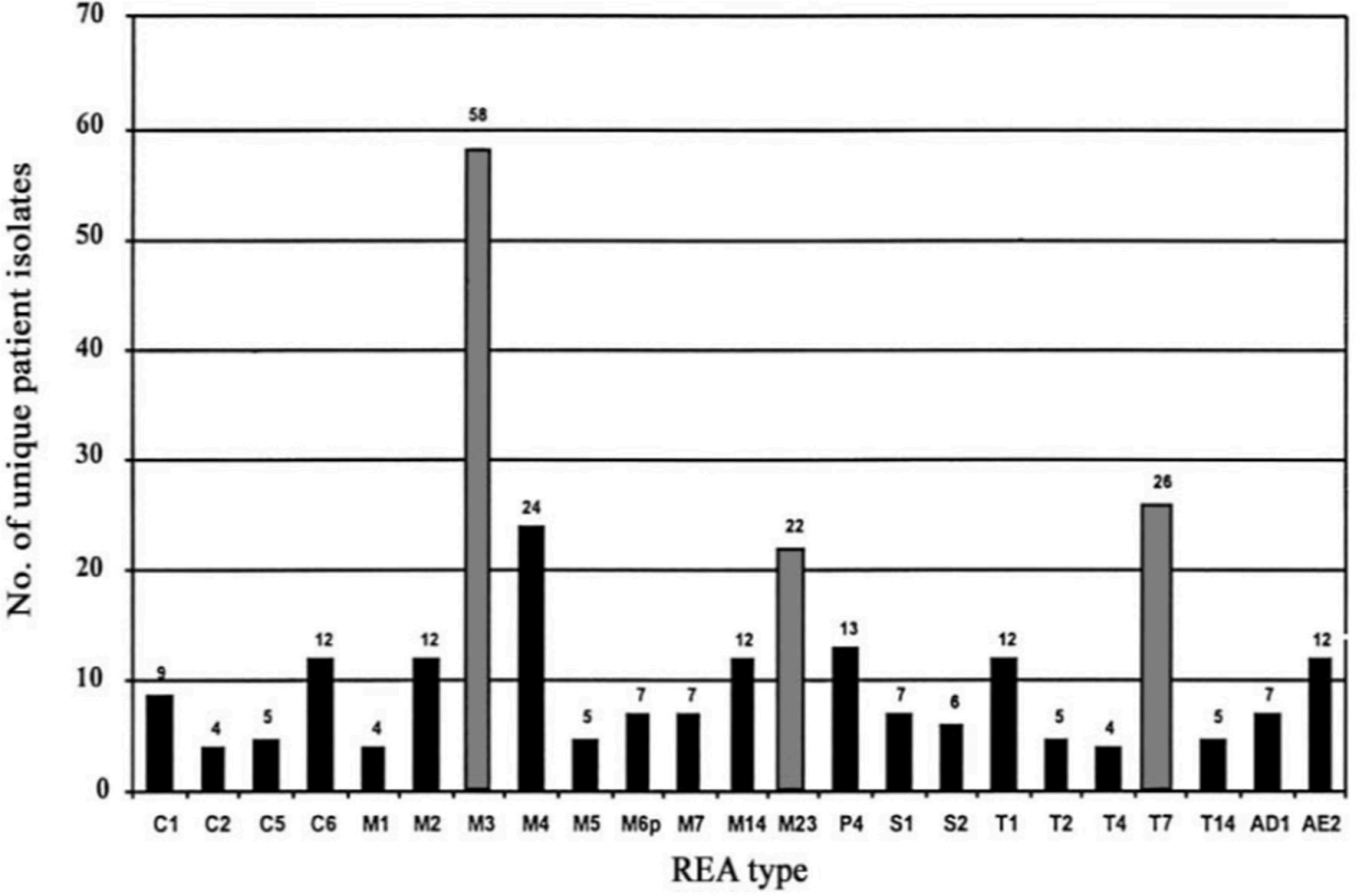
Frequency of isolation of *Clostridium difficile* nontoxigenic restriction endonuclease analysis (REA) types from individual patients during 1982–1993. Gray bars indicate frequency of REA types used in hamster prevention trials. From Sambol et al. (2002) with permission.

**Figure 3:**
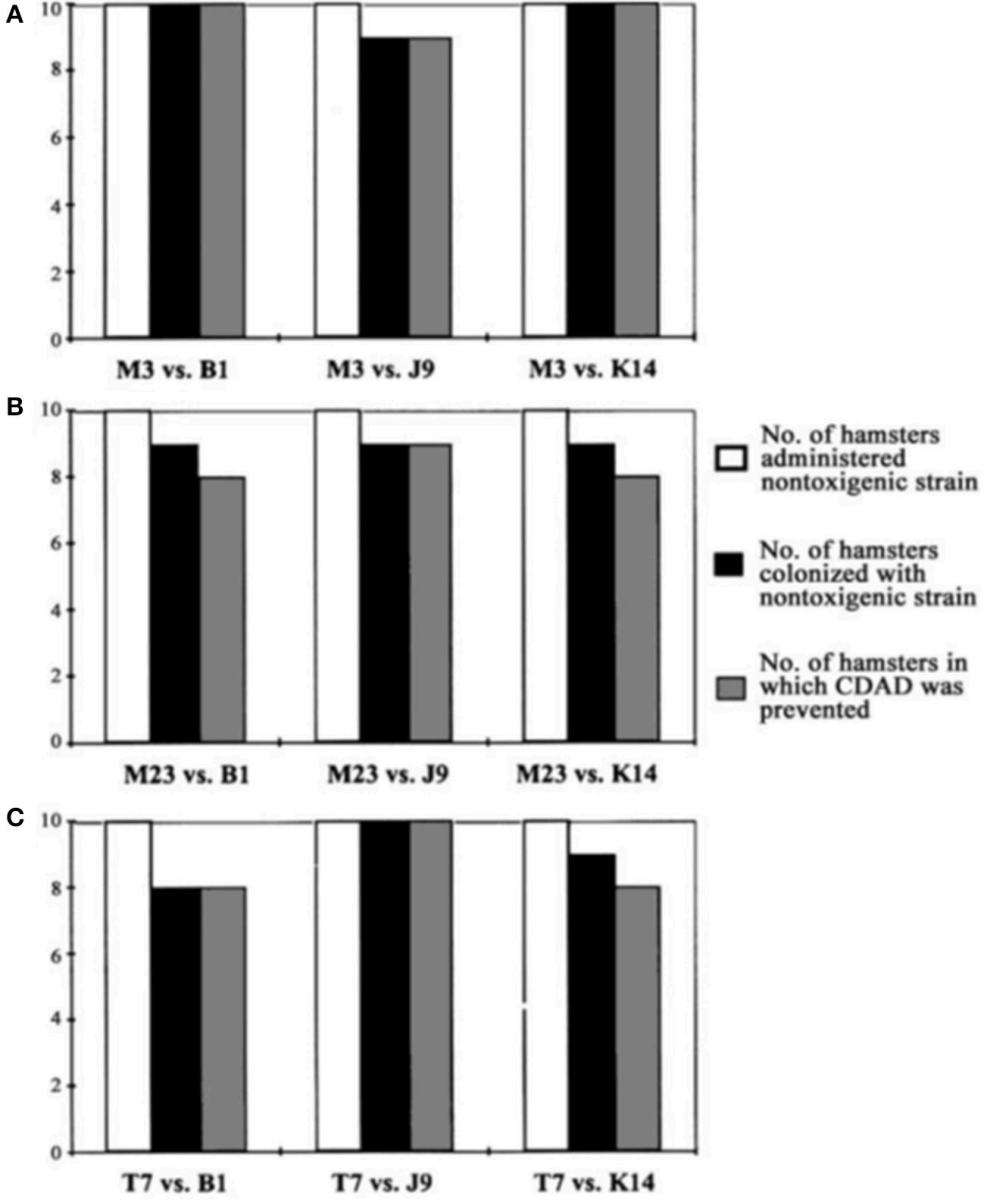
Colonization by nontoxigenic restriction endonuclease analysis (REA) types and prevention of *Clostridium difficil*e-associated disease (CDAD) in hamsters. Bars indicate number of animals colonized by nontoxigenic *C. difficile* REA type M3 **(A)**, type M23 **(B)**, and type T7 **(C)** and challenged with 3 different toxigenic *C. difficile* REA types (B1, J9, and K14). From Sambol et al. (2002) with permission.

To test the effectiveness of NTCD as a preventive of infection with toxigenic *C. difficile*, the above described hamster model was used (Sambol et al., 2002). Three NTCD strains were selected from among isolates collected with rectal swabs from asymptomatic hospitalized patients; M3, M23, and T7, all of which were isolated at high frequency from patients (Figure 2). The NTCD inoculum was 10^6^ spores by oral gavage on day 2 following clindamycin 30 mg/kg orally. Toxigenic *C. difficile* challenge with strains B1, J9, or K14 with 100 spores by oral gavage occurred on day 5 following clindamycin (3 days after the NTCD inoculum). Prevention of CDI mortality ranged from 87 to 97% (Figure 3) and correlated closely with detection of NTCD colonization of the stool prior to challenge with toxigenic *C. difficile* strains. Colonization with NTCD persisted in these surviving hamsters until end of study which varied from 55 to 106 days and there was no late mortality from toxigenic *C. difficile*.

#### NTCD prevention durability

Durability of protection against late challenge with toxigenic *C. difficile* was performed with M3 and M23 NTCD. In the first of these studies M3 colonized 5 of 5 hamsters on day 4 following inoculation on day 2 following clindamycin. All 5 hamsters lost colonization between days 23 and 44. The uncolonized hamsters were challenged with 100 spores of toxigenic B1 *C. difficile* on day 62 and survived until day 153 end of study. In a second M3 experiment 3 of 4 hamsters were colonized on day 3 following inoculation on day 2 post clindamycin and were challenged on day 63 with 100 spores of toxigenic J9 and all survived with 3 of 4 remaining colonized with M3 until end of study day 125. In a third experiment, 12 hamsters were given clindamycin on day 0; 10 received NTCD strain M23 on day 2 and became colonized by day 5. All 12 hamsters were challenged with toxigenic strain B1 on day 41 and all survived (10 colonized with M23 and 2 uncolonized). All 10 remained colonized until day 49 when the study ended. These studies suggest that hamsters apparently recover colonization resistance following clindamycin by days 41–62 and are protected from late challenge with toxigenic *C. difficile* whether colonized with NTCD or not.

Patients are occasionally treated with a single antibiotic dose, as for example when undergoing surgical prophylaxis, and would be representative of the experiments with hamsters following a single dose of antibiotic. However, most infection treatment is carried out using multiple doses, often on the same day and for multiple days at a time. This raises the question of how NTCD might be employed to prevent CDI since many of the antibiotics used systemically will be active against NTCD and presumably prevent it from colonizing the patient. NTCD REA types M3, M23, and T7 are susceptible to most antibiotics including ampicillin, clindamycin, erythromycin, tetracycline, trimethoprim-sulfamethoxazole and metronidazole, but resistant to ciprofloxacin and ceftriaxone (Sambol et al., 2002), patterns that are typical of most *C. difficile* isolates. Resistance to clindamycin, tetracyclines, carbapenems and erythromycins as well as very high-level resistance to fluoroquinolones is known to occur in *C. difficile* and may be transferrable. Whereas use of a highly antibiotic resistant strain of NTCD would favor more frequent colonization, it would also increase the risk of antibiotic resistance transfer to other bacteria in the gastrointestinal tract.

#### Effect of daily clindamycin and resistance in NTCD

To assess the effect of antibiotic resistance of NTCD on colonization and prevention of infection by toxigenic *C. difficile*, Merrigan et al. (2003) conducted experiments in hamsters treated with daily oral doses (30 mg/kg) of clindamycin for 5 consecutive days. Two strains of NTCD, REA type M3 (clindamycin susceptible at 0.5 mcg/ml), REA type M13 (clindamycin resistant at >256 mcg/ml) and a toxigenic strain of *C. difficile*, REA type B1 (clindamycin resistant at >256 mcg/ml) were used. Clindamycin resistance in type M13 and B1 was determined to be due to presence of the *erm*(B) gene. M3 was negative for *erm*(B). Colonization experiments with M3 and M13 were conducted in groups of 5 hamsters by administering (1) a single dose of 1 × 10^6^ spores on day 3 of clindamycin, (2) a daily dose of 1 × 10^6^ spores on days 3–5 of clindamycin or (3) a daily dose of 1 × 10^6^ spores on days 3–7. Colonization occurred with all doses of resistant strain M13 within 1.25 days of the first dose. No colonization occurred following the single dose of susceptible strain M3, but following the 3-day and 5-day administrations all 10 hamsters eventually became colonized but after a mean lag time of 6.4 to 6.6 days following the first dose. When the experiment was repeated with challenge with toxigenic strain B1, 100 spores orogastrically, on day 5, day 7, or day 9, all hamsters were protected by all doses of clindamycin resistant M13. Clindamycin susceptible M3 gave no protection against challenge with B1 on day 5, but protected 1 of 5 hamsters challenged on day 7, and 5 of 5 challenged on day 9. The results of the experiment clearly show the advantage of an antibiotic resistant NTCD strain in preventing CDI, but also suggest that an antibiotic susceptible strain can be effective if administered daily past the end of antibiotic administration, albeit without as many days of prevention due to the longer time required to establish colonization in the presence of an antibiotic to which it is susceptible. Obviously, a toxigenic *C. difficile* strain resistant to the antibiotic being given has an advantage over susceptible NTCD while the antibiotic is being given, an advantage that is lost within a few days of stopping the antibiotic.

#### NTCD prevention after daily exposure to antibiotics other than clindamycin

**Figure 4:**
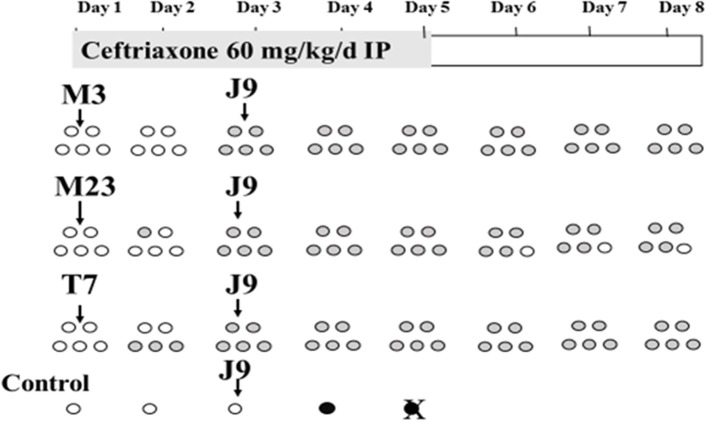
Colonization of non-toxigenic *Clostridium difficile* strains M3, M23, or T7 and subsequent challenge with toxigenic strain J9 during daily ceftriaxone administration. Animals are indicated by ovals: open ovals indicate no detection of *C. difficile* in stool; gray-shaded ovals indicate detection of non-toxigenic *C. difficile* in stool; black ovals indicate detection of toxigenic strain J9 in stool. An X superimposed over an oval indicates the day of death. IP, intraperitoneally. From Merrigan et al. (2009).

Similar experiments were conducted to determine NTCD protection during continuous daily administration of ampicillin or ceftriaxone (Merrigan et al., 2009) with the exception that the dose of the toxigenic challenge strain was raised to 1 × 10^6^ spores to further test NTCD prevention. Most strains of *C. difficile* are resistant to cephalosporins including ceftriaxone, and are susceptible to penicillins such as ampicillin. In the ceftriaxone experiments the antibiotic was given intraperitoneally (60 mg/kg) daily for 5 days and NTCD strains M3, M23, or T7 were given to groups of 5 hamsters orogastrically at a dose of 1 × 10^6^ spores 3 h following the first ceftriaxone dose. The toxigenic challenge strain (REA type J9, 1 × 10^6^ spores orogastrically) was responsible for multiple outbreaks of CDI in U.S. hospitals in the 1990s (Johnson et al., 1999). All the strains used were resistant to ceftriaxone; M3 and M23 MIC = 128 mg/mL; T7 MIC = 96 mg/mL; and J9 MIC = >256 mg/mL. Results are shown in Figure 4 and demonstrate colonization within 48h for all NTCD strains and protection from J9 challenge on day 3 in all hamsters. A single control animal died within 48h as expected with J9 challenge.

**Figure 5:**
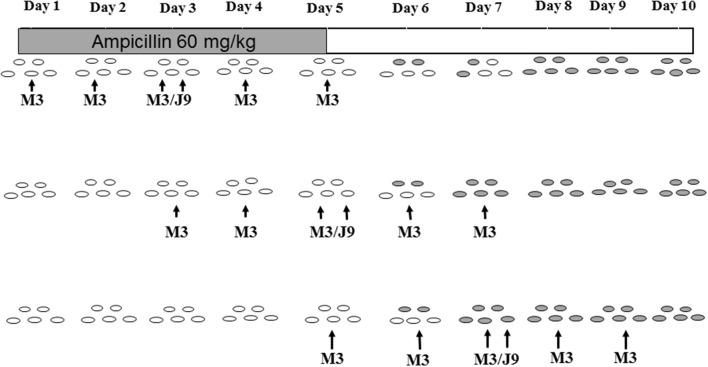
Colonization with non-toxigenic *Clostridium difficile* M3 and challenge with toxigenic strain J9 during daily oral ampicillin administration for 5 days. Both J9 and M3 are susceptible to ampicillin. Animals are indicated by ovals: open ovals indicate no detection of *C. difficile* in stool; gray-shaded ovals indicate detection of non-toxigenic M3 in stool. PO, orally. From Merrigan et al. (2009).

Protection against daily administration of an antibiotic such as ampicillin to which NTCD and toxigenic *C. difficile* are both susceptible poses a different challenge (Merrigan et al., 2009). In these experiments NTCD REA type M3 (ampicillin MIC = 2.0 mcg/ml) was utilized for prevention (1 × 10^6^ spores daily) and challenged at intervals of 2 days with 1 × 10^6^ spores of toxigenic strain J9 (ampicillin MIC = 0.75 mcg/ml). Ampicillin was given orogastrically (60 mg/kg daily) to groups of 4 hamsters for 5 days. As expected based on pilot studies, neither M3 or J7 colonized during ampicillin administration, but were able to establish colonization by day 8 if given near the end of the ampicillin course, indicating that following fecal excretion of the inhibitory antibiotic colonization could be established. Experiments were then designed to determine if NTCD M3 administered daily for 5 days to 3 groups of 4 hamsters beginning on day 1, day 3, and day 5 could protect against toxigenic J9 challenges on the middle day of each 5-day M3 course, days 3, 5, and 7, with both strains administered orogastrically at 1 × 10^6^ spores. The study was designed to simulate periodic exposure to toxigenic *C. difficile* while being given NTCD daily. The experiment is shown schematically in Figure 5 and demonstrates absence of J9 infection despite no colonization by either strain during the 5 days of ampicillin, followed by progressive colonization by M3 on days 7 and 8 in all hamsters. Results of these experiments suggest that in the presence of daily administration of an antibiotic to which NTCD is susceptible, that NTCD should be given daily and for several days after cessation of the antibiotic to attain successful colonization. Toxigenic *C. difficile* is not likely to infect during antibiotic administration and daily NTCD prophylaxis unless the toxigenic strain is resistant to the antibiotic and NTCD is susceptible.

#### NTCD prevention against the highly virulent BI strain of C. difficile

Early in the twenty first century it became apparent that CDI rates, morbidity and mortality were increasing in North America and were attributed at least in part to the presence of an epidemic strain characterized as BI group by REA, NAP1 by pulsed field gel electrophoresis, and 027 by PCR ribotyping and referred to as NAP1/BI/027 (Loo et al., 2005; McDonald et al., 2005). This strain spread throughout the United States and Canada as well as the United Kingdom and much of Europe as CDI rates rose precipitously in these countries. NAP1/BI/027 carries a third toxin, binary toxin and when compared to historic isolates of the same type it was apparent that high-level fluoroquinolone resistance had been acquired (McDonald et al., 2005). This prompted study of these strains in the hamster model by Razaq et al. (2007) who compared epidemic BI strains BI6 and BI17 to historic non-epidemic BI strain BI1 and 2 standard toxigenic strains, K14 and 630 (REA type R23). In these studies groups of 10 hamsters were given 100 spores of each strain of *C. difficile* orogastrically 5 days following a single clindamycin dose (30 mg/kg orally). Although group BI strains were not more rapidly fatal than standard toxinotype 0 strain K14, they were more rapidly fatal than strain 630 and epidemic strain BI6 was the most rapidly fatal of all the strains tested.

**Figure 6:**
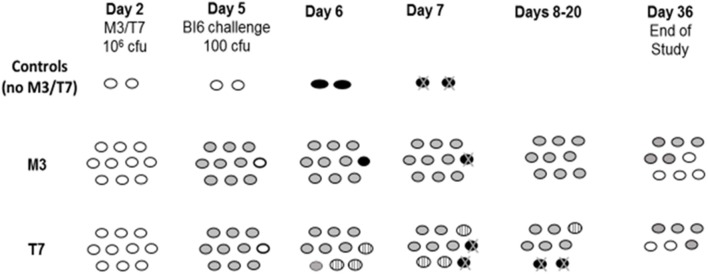
Hamsters (*n* = 10/group) challenged with epidemic toxigenic BI6 *Clostridium difficile*. Day 2 = 2 days post-clindamycin treatment. *White ovals*; uncolonized hamsters. *Gray ovals*; non-toxigenic colonized hamsters. *Striped ovals*; hamsters co-colonized with non-toxigenic and toxigenic *C. difficile. Black ovals with* “*X*”; hamster death from toxigenic BI6. From Nagaro et al. (2013).

The widespread clinical presence of epidemic strain NAP1/BI/027 raised the question of whether NTCD strains would be effective in preventing disease due to BI strains in the hamster model. Nagaro et al. (2013) gave groups of 12 hamsters oral clindamycin (30 mg/kg orally) followed 2 days later by 1 × 10^6^ spores of NTCD strains M3 or T7 in 10 hamsters, and on day 5 challenged all 12 with 100 spores of historic strain BI1 or epidemic strain BI6. All hamsters colonized with M3 or T7 and were protected against challenge with historic strain BI1. NTCD strain M3 colonized 9 of 10 hamsters and protected against BI6 challenge in the 9 colonized hamsters, but not the uncolonized animal. However, epidemic strain BI6 proved more difficult for protection by NTCD strain T7. For the first time in these hamster experiments we found NTCD colonization displaced by the toxigenic strain (Figure 6). Five of 10 hamsters were co-colonized with T7 and BI6 on day 7 and 4 of the 5 died. The remaining co-colonized hamster lost BI6 colonization on day 21 and survived. These results suggest that toxigenic and NTCD strains of *C. difficile* compete for colonization and that certain strains such as BI6 (but not its historic predecessor BI1) despite their lower inoculum and later administration are able to compete more effectively than other toxigenic strains against NTCD strain T7, but not against strain M3. Thus, differences in colonization efficiency are evident for both toxigenic strains of *C. difficile* as well as NTCD strains.

#### Possible mechanism of NTCD prevention

The mechanism by which *C. difficile* strains establish colonization is not established. One possibility is that strains differ in their ability to adhere to colonic mucosal cells or mucus. Merrigan et al. (2013) utilized a derivative of the Caco-2-derived human intestinal epithelial cell-line to assess adherence of 33 strains of toxigenic *C. difficile* (with an emphasis on epidemic NAP1/BI/027 and ribotype 078 strains) and 3 NTCD strains (REA types M3, M23, and T7) in this assay with a focus on differences in surface layer protein A (SlpA) in the strains. The average adherence range of vegetative cells was approximately 8–11% for strain 630, 2–4% for strain K14, 5–9% for strain BI17, 8–14% for NTCD strain M3, and 9–12% for NTCD strain T7. As a group, BI/NAP1/027 strains exhibited a mean adherence value of 4.6%. Further experiments showed that pretreatment of cell layers with surface layer protein (SLP) preparations inhibited *C. difficile* adherence up to 80% with the maximum SLP dose. SLP inhibition was not strain specific; NTCD M3 SLP inhibited M3 and BI17 adherence equally, and BI17 SLP inhibited BI17 and M3 adherence equally. Sequence analysis of the SlpA proteins for both the high molecular weight and low molecular weight subunits was compared among strains. One might postulate that there would be a high degree of conservation of amino acid sequences among highly competitive strains, yet NTCD strain M3 displayed the least sequence conservation, especially in the low molecular weight subunit, compared not only to epidemic toxigenic strains, but also when compared to NTCD strain T7.

#### NTCD prevention of recurrent CDI

**Figure 7:**
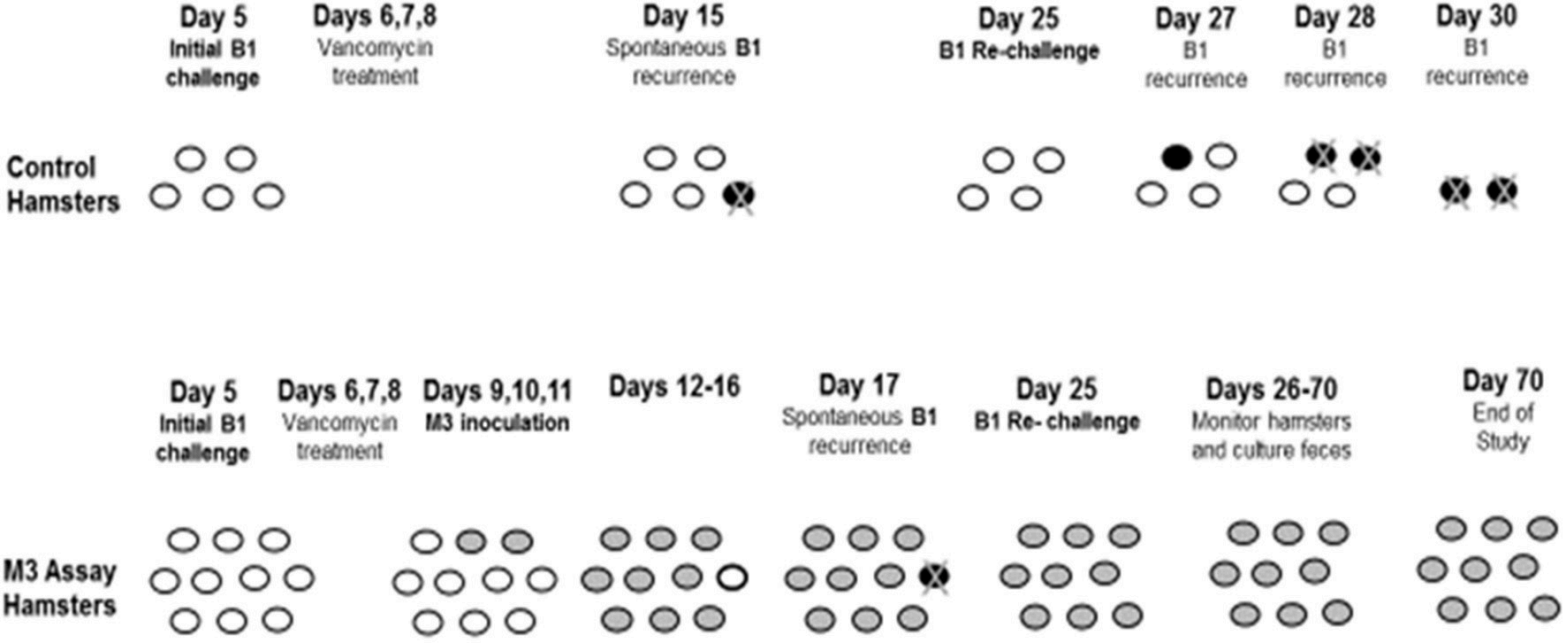
Prevention of recurrence with NTCD-M3 following vancomycin treatment of toxigenic B1 *C. difficile* infection in hamsters. Day 5 = 5 days post-clindamycin treatment. *White ovals*: uncolonized hamsters. *Black ovals*: toxigenic infected hamsters. *Gray ovals*: non-toxigenic colonized hamsters. *Black ovals with* “*X*”: hamster death from toxigenic B1. From Sambol et al. (2004).

Patients who have CDI incur about a 20–30% risk of having a recurrence of their symptoms following successful treatment. If such patients could be colonized with NTCD following antibiotic treatment of CDI, it could prevent subsequent recurrence. To test this possibility, the CDI treatment model in hamsters that demonstrated late recurrence of CDI mortality following treatment with vancomycin was modified to administer NTCD M3 following vancomycin treatment (Swanson et al., 1991; Sambol et al., 2004). Sambol et al. (2004) gave a group of 15 hamsters oral clindamycin (30 mg/kg) followed 5 days later by 1 × 10^4^ spores of epidemic strain B1. Vancomycin was administered orogastrically in doses of 100 mg/kg daily for 3 consecutive days on Days 6, 7, and 8 with the first dose administered within 14 h of the initial B1 challenge. On Days 9, 10, and 11, NTCD strain M3 was orally administered to 10 of the 15 hamsters at 1 × 10^6^ spores/day per hamster (5 control hamsters did not receive NTCD). On Day 25, all surviving hamsters were given a re-challenge dose of 1 × 10^4^ toxigenic B1 spores to simulate re-exposure to the toxigenic strain (Figure 7). Of the control hamsters, one out of 5 was infected with B1 three days after vancomycin treatment, and died on Day 15, a spontaneous B1 recurrence. After the B1 re-challenge on Day 25, 4 of 4 surviving control hamsters became infected with B1 between Days 27 and 30, and died by Day 30.

Of the hamsters given NTCD, 9 of 10 colonized with M3 between Days 10–16; one hamster remained uncolonized until Day 17, when it became infected with epidemic strain B1 and died, a spontaneous CDI recurrence. After the B1 re-challenge on Day 25, all 9 surviving hamsters remained colonized with M3 until study end on Day 70 with no evidence of B1 infection. These results demonstrate that NTCD colonization following vancomycin treatment is successful and will prevent recurrent *C. difficile* infection.

#### Potential for NTCD acquisition of toxin genes

Concern has been raised that in clinical use NTCD strains may acquire the toxin A and B pathogenicity locus (PaLoc) and become pathogenic. Brouwer et al. (2013) have shown *in vitro* that toxigenic strain 630Δerm can transfer the PaLoc to NTCD strain CD37, which is REA type T18. PaLoc-containing transconjugants were obtained at a frequency of 7.5 × 10^−9^ transconjugants per donor in filter matings. DNA fragments containing the PaLoc ranged in size from 66,034 to 272,977 bp. Transfers from 630Δerm to two other NTCD isolates, PCR ribotypes 138 and 140, were also successful demonstrating that PaLoc transfer is not restricted to a specific donor-recipient pair. Supernatants of recipient strain CD37 following PaLoc transfer were found to cause cell rounding in the cell cytotoxicity assay that was neutralized by toxin B antibodies indicating that the PaLoc transfer resulted in functional toxin production. The mechanism by which PaLoc transfer takes place has not been precisely defined, but transfer via a cell-to-cell conjugation-like transfer mechanism has been proposed (Brouwer et al., 2016). The likelihood of such transfers occurring *in vitro* is unknown, but would presumably require high concentrations of a toxigenic *C. difficile* strain and NTCD in close proximity in the gastrointestinal tract of a host. In the hamster model, we have demonstrated that vancomycin administered daily for 3 days will eliminate NTCD detection in stool, and would presumably be effective in managing CDI if PaLoc transfers were to occur in patients.

### Back again to bedside: clinical trials

The above pre-clinical data were used to file investigational new drug applications with agencies in the US, Canada and Europe. Manufacturing of spores of NTCD-M3 under cGMP was undertaken successfully by ViroPharma, Inc. and the spores prepared in liquid suspension for safety testing in healthy volunteers at doses of 10^4^, 10^6^, and 10^8^ spores/dose or placebo (Villano et al., 2012). NTCD-M3 was found to be safe in younger volunteers age 18–45 years at all three single doses given in the absence of any antibiotic pre-treatment. To better simulate the likely age of patients with CDI, volunteers age >60 years (age range 60–73 years) were given single doses of 10^4^, 10^6^, and 10^8^ spores or placebo safely. Volunteers >60 years were then given the highest dose, 10^8^ spores twice daily for 5 days and tolerated this safely. This was the only group in which NTCD could be detected in the stools of the volunteers in the absence of prior antibiotic administration. Stool cultures first became positive on days 2–4 and were positive in 6 of 8 volunteers at day 7 but in none at day 14 and 21 suggesting the possibility that this may have been pass-through of NTCD spores and not colonization.

Finally, groups of 12 >60 year old volunteers were pre-treated with 5 days of oral vancomycin 125 mg 4 × daily to simulate patients being treated for CDI to determine if colonization with NTCD would occur post vancomycin treatment. Each group of 9 NTCD and 3 placebo subjects was given 10^4^ or 10^6^ or 10^8^ spores of NTCD-M3 or placebo daily for 14 days beginning the day following the last vancomycin dose. In each group the 9 volunteers given NTCD-M3 had NTCD detected in their stools during the 14-day administration and in 4 to 5 volunteers on days 21 and 28 following NTCD. In the 3 volunteers receiving placebo in the 10^4^ and 10^6^ NTCD-M3 spore cohorts one volunteer in each cohort became colonized with a toxigenic strain of *C. difficile* but remained asymptomatic. In the cohort receiving 10^8^ spores of NTCD-M3 the 3 placebo patients all became colonized with NTCD-M3, indicating that at this highest dose of NTCD-M3 there was transmission of NTCD-M3 from the active recipient group or the environment to the placebo group who were housed on the same clinical trial facility. No safety issues were identified in the volunteers receiving NTCD-M3.

**Figure 8:**
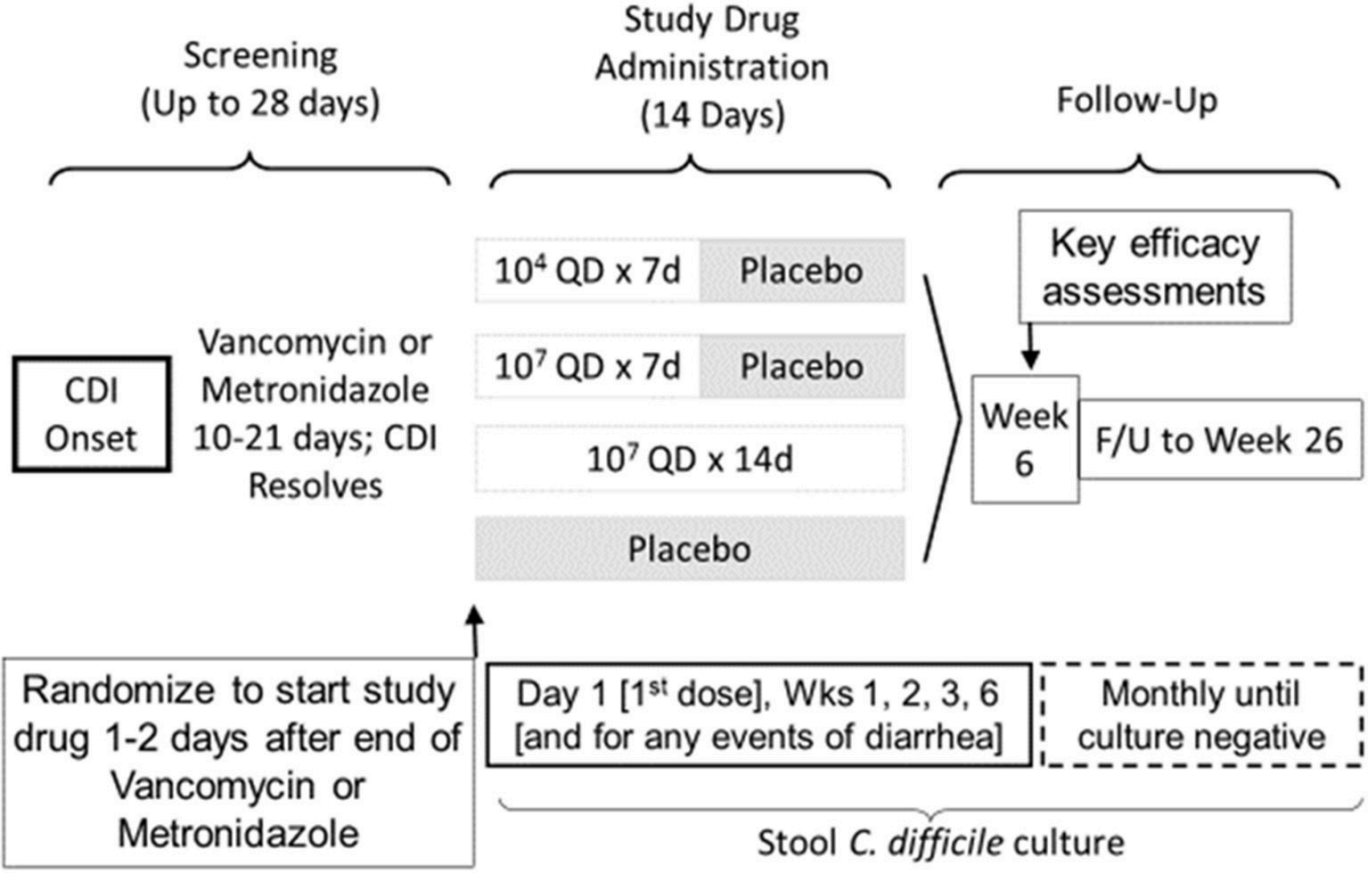
Trial Design for Phase 2 clinical trial of study drug NTCD-M3, spores of non-toxigenic *C. difficile* strain M3 (Gerding et al., 2015).

For the first time since Seal et al. (1987) reported the treatment of 2 CDI patients with NTCD in 1987, NTCD was again administered in a Phase 2 double blind, randomized, dose-ranging, placebo controlled trial of NTCD for prevention of recurrent CDI in patients who have had their first episode or first recurrence of CDI. The primary outcome of the study was safety, with fecal colonization rate, CDI recurrence rate, and optimal dosing of NTCD-M3 as exploratory secondary outcomes. The study design shown in Figure 8 compared 2 doses, 10^4^ and 10^7^ spores given orally in liquid once daily for 2 durations, 7 days (10^7^ spores) and 14 days (10^4^ and 10^7^ spores) compared to placebo for 14 days. NTCD-M3 was begun 1–2 days following completion of successful treatment of CDI with vancomycin or metronidazole (Gerding et al., 2015). A total of 168 patients were randomized and treated and 157 completed treatment. The four arms of the study were well balanced in terms of patient age (range 18–94 years) with 39% >65 years old. Most patients were having their primary CDI episode (83%), were outpatients (76%), and were treated with metronidazole (60%). Vancomycin was used for treatment in 20% of subjects and 20% were treated with both vancomycin and metronidazole.

**Figure 9:**
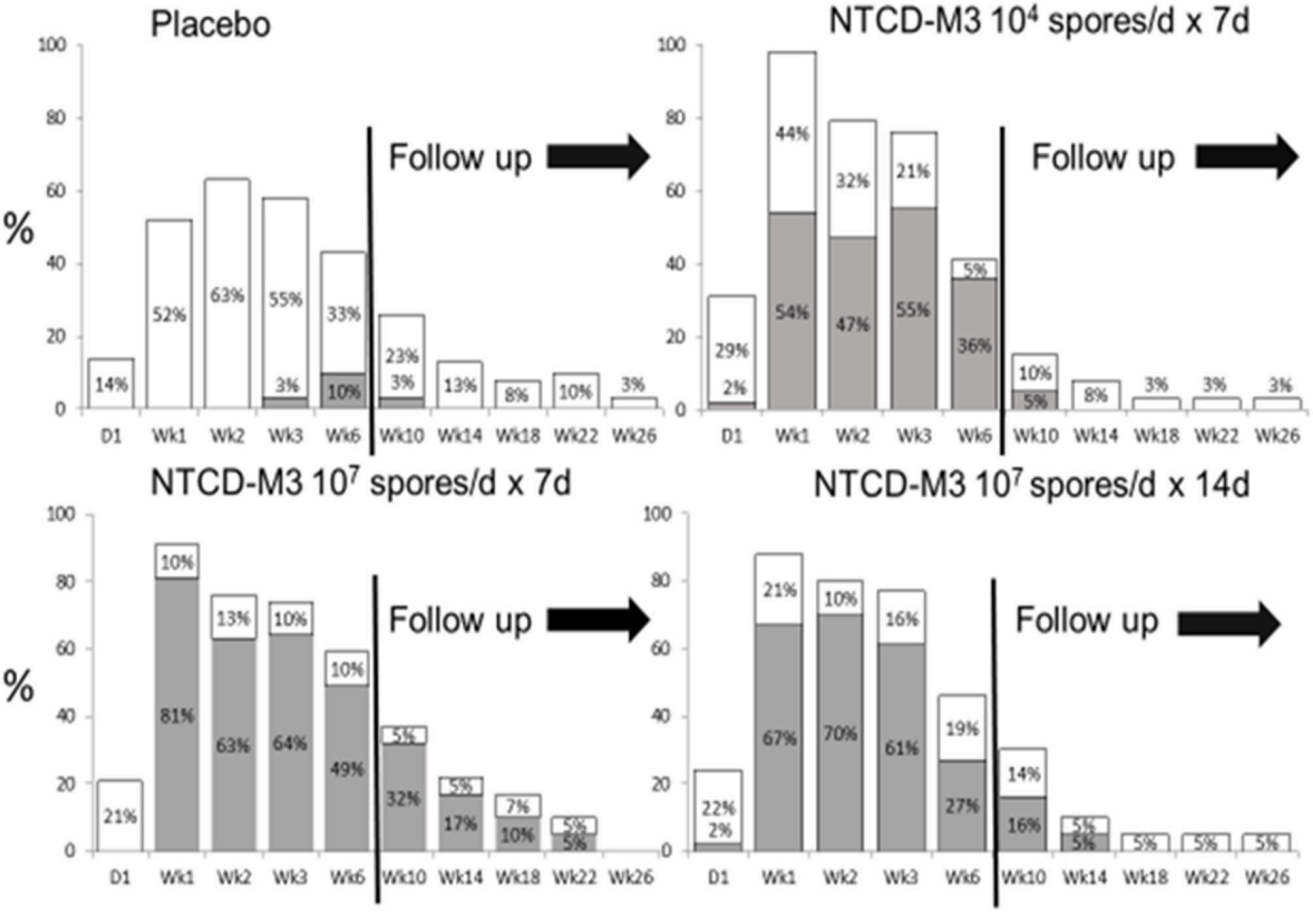
Proportion of patients with positive *Clostridium difficile* stool cultures at specified visits through week 6 and during follow-up (weeks 10–26) by treatment group for the intent-to-treat safety population. White bars: culture positive for toxigenic *C. difficile*. Gray bars: culture positive for NTCD. NTCD, non-toxigenic *C. difficile*. From Gerding et al. (2015) (Supplementary Material) with permission.

Safety results indicated that participants who received NTCD-M3 had fewer treatment emergent adverse events and fewer serious adverse events than placebo patients with the exception of headache which occurred in 10% of NTCD-M3 recipients and 2% of placebo (Gerding et al., 2015). No serious adverse events were considered treatment related. Colonization by NTCD-M3 was defined as detection of NTCD-M3 in stool at any time following completion of treatment. Figure 9 demonstrates that colonization with toxigenic *C. difficile* following antibiotic treatment of CDI is common (63% of placebo subjects at week 2) whereas with administration of NTCD-M3, much of the toxigenic *C. difficile* colonization was replaced by NTCD-M3; 71% of participants who received 10^7^ spores and 63% who received 10^4^ spores. Colonization with NTCD-M3 was maximal in weeks 1–3 and slowly declined thereafter until last detected in week 22. A small number of participants still harbored toxigenic *C. difficile* at 26 weeks, the last time stools were sampled.

Prevention of recurrence of CDI is summarized in Table 1. Using the protocol definition of recurrence of CDI at 6 weeks, recurrence rate was 13 (30%) of 43 placebo subjects and 14 (11%) of 125 NTCD-M3 subjects (odds ratio [OR], 0.28; 95%CI, 0.11–0.69; *P* = 0.006); the lowest recurrence was in 2 of 43 (5%) patients receiving 10^7^ spores/day for 7 days (OR, 0.1; 95%CI, 0.0–0.6; *P* = 0.01 vs. placebo). Using an alternate definition of recurrent CDI defined as use of antibacterial treatment for CDI, the recurrence rate was 14 of 43 (33%) placebo subjects and 17 of 125 (14%) NTCD-M3 subjects (*p* = 0.009). NTCD-M3 also reduced all-cause diarrhea from 77% in placebo subjects to 57% of NTCD-M3 subjects (*p* = 0.020). There was good correlation of CDI prevention with colonization by NTCD-M3; among subjects who received NTCD-M3 and became colonized, the CDI recurrence rate was 2 of 86 (2%) vs. 12 of 39 (31%) subjects who received NTCD-M3 and were not colonized (*p* < 0.001). Dose-ranging indicated that 10^7^ spores were more effective than 10^4^ spores, and there was no advantage to extending treatment to 14 days vs. 7 days for the 10^7^ dose. In summary, NTCD-M3 was safe and effective in preventing CDI in patients who were experiencing their first CDI episode or first recurrence of CDI.

**Table 1:** *C. difficile* recurrence and any diarrhea event by treatment arm in a phase 2 clinical trial of NTCD-M3 administered at two different doses of 10^4^ and 10^7^ spores for 7 or 14 days vs. placebo for 14 days (Gerding et al., 2015).

		**Placebo**	**10^4^ × 7d**	**10^7^ × 7d**	**10^7^ × 14d**	**All NTCD**
Number of patients, n		43	41	43	41	125
CDI recurrence, n (%)		13 (30)	6 (15)	2 (5)	6 (15)	14 (11)
	*p*-value^*^		0.11	0.01	0.10	0.006
CDI recurrence defined by antibacterial use for CDI treatment, n (%)		14 (33)	6 (15)	4 (9)	7 (17)	17 (14)
	*p*-value^*^		0.07	0.02	0.14	0.009
Any event of diarrhea (of any severity) or CDI, n (%)		33 (77)	23 (56)	25 (58)	23 (56)	71 (57)
	*p*-value^*^		0.05	0.09	0.02	0.020

* *p-values adjusted for pre-specified parameters: use of metronidazole vs. vancomycin, and primary episode vs. first recurrence*.

## Summary

NTCD has a long history of basic laboratory and clinical experience that suggests it is a safe and very effective preventive for recurrent CDI (hamster and human data) and as a preventive for first episode CDI if used in subjects receiving antibiotics (hamster data only). Further development is required with phase 3 clinical trials for recurrent CDI prevention and for phase 2 and 3 primary CDI prevention clinical trials.

## Author contributions

DG wrote the first manuscript draft and designed the experiments described from his laboratory and provided funding. SJ and SS edited the manuscript, contributed text and figures, and were instrumental in designing and carrying out the described experiments in the DG laboratory.

### Conflict of interest statement

DG holds patents (unlicensed, no royalties) and technology for the use of NTCD for prevention of CDI and is Scientific Advisory Board Member for Rebiotix, Actelion, Merck, Summit and DaVolterra, is a consultant for Pfizer, MGB Pharma, and Sanofi Pasteur, and holds a research grant from Seres Therapeutics. SJ is Scientific Advisory Board Member for Bio-K+, Synthetic Biologics, Summit and CutisPharma. The remaining author declares that the research was conducted in the absence of any commercial or financial relationships that could be construed as a potential conflict of interest.
